# Flavin Oxidase-Induced ROS Generation Modulates PKC Biphasic Effect of Resveratrol on Endothelial Cell Survival

**DOI:** 10.3390/biom9060209

**Published:** 2019-05-30

**Authors:** Anna Maria Posadino, Roberta Giordo, Annalisa Cossu, Gheyath K. Nasrallah, Abdullah Shaito, Haissam Abou-Saleh, Ali H. Eid, Gianfranco Pintus

**Affiliations:** 1 Department of Biomedical Sciences, School of Medicine, University of Sassari, CAP 07100 Sassari, Italy; posadino@uniss.it (A.M.P.); cossuannalisa@libero.it (A.C.); 2 Biomedical Research Center, Qatar University, Doha P.O. Box 2713, Qatar; roberta.giordo@qu.edu.qa (R.G.); gheyath.nasrallah@qu.edu.qa (G.K.N.); 3 Department of Biomedical Sciences, College of Health Sciences, Qatar University, Doha P.O. Box 2713, Qatar; 4 Department of Biological and Chemical Sciences, Faculty of Arts and Sciences, Lebanese International University, 1105 Beirut, Lebanon; Abdallah.shaito@liu.edu.lb; 5 Department of Biological and Environmental Sciences, College of Arts and Sciences, Qatar University, Doha P.O. Box 2713, Qatar; hasaleh@qu.edu.qa; 6 Department of Pharmacology and Toxicology, American University of Beirut, Beirut P.O. Box 11-0236, Lebanon

**Keywords:** ROS, resveratrol, endothelial cells, anti- and pro-oxidant effect, dose-dependence, cell damage, PKC, flavin oxidase

## Abstract

Background: Dietary intake of natural antioxidants is thought to impart protection against oxidative-associated cardiovascular diseases. Despite many in vivo studies and clinical trials, this issue has not been conclusively resolved. Resveratrol (RES) is one of the most extensively studied dietary polyphenolic antioxidants. Paradoxically, we have previously demonstrated that high RES concentrations exert a pro-oxidant effect eventually elevating ROS levels leading to cell death. Here, we further elucidate the molecular determinants underpinning RES-induced oxidative cell death. Methods: Using human umbilical vein endothelial cells (HUVECs), the effect of increasing concentrations of RES on DNA synthesis and apoptosis was studied. In addition, mRNA and protein levels of cell survival or apoptosis genes, as well as protein kinase C (PKC) activity were determined. Results: While high concentrations of RES reduce PKC activity, inhibit DNA synthesis and induce apoptosis, low RES concentrations elicit an opposite effect. This biphasic concentration-dependent effect (BCDE) of RES on PKC activity is mirrored at the molecular level. Indeed, high RES concentrations upregulate the proapoptotic *Bax*, while downregulating the antiapoptotic *Bcl-2*, at both mRNA and protein levels. Similarly, high RES concentrations downregulate the cell cycle progression genes, *c-myc*, ornithine decarboxylase *(ODC)* and cyclin D1 protein levels, while low RES concentrations display an increasing trend. The BCDE of RES on PKC activity is abrogated by the ROS scavenger Tempol, indicating that this enzyme acts downstream of the RES-elicited ROS signaling. The RES-induced BCDE on HUVEC cell cycle machinery was also blunted by the flavin inhibitor diphenyleneiodonium (DPI), implicating flavin oxidase-generated ROS as the mechanistic link in the cellular response to different RES concentrations. Finally, PKC inhibition abrogates the BCDE elicited by RES on both cell cycle progression and pro-apoptotic gene expression in HUVECs, mechanistically implicating PKC in the cellular response to different RES concentrations. Conclusions: Our results provide new molecular insight into the impact of RES on endothelial function/dysfunction, further confirming that obtaining an optimal benefit of RES is concentration-dependent. Importantly, the BCDE of RES could explain why other studies failed to establish the cardio-protective effects mediated by natural antioxidants, thus providing a guide for future investigation looking at cardio-protection by natural antioxidants.

## 1. Introduction

Reactive oxygen species (ROS) are byproducts of regular aerobic metabolism. Although widely recognized as harmful entities, depending on their concentration, ROS may exert different effects on cell physiology. ROS levels produced under physiological conditions are the result of well-orchestrated balancing activities of ROS-generating enzymes and endogenous antioxidant mechanisms. These ROS level balancing activities modulate several signalling cascades implicated in normal physiological responses [1]. On the other hand, when the above-mentioned balance is lost, the levels of ROS increase dramatically, and a state of oxidative stress is established causing damage to cellular structures and internal components [2,3]. Consequently, ROS plays a critical role in both physiological and pathophysiological processes [4].

Redox-regulated signalling pathways are common ROS-mediated signal transduction mechanisms widely adopted by the cells [5]. ROS can modulate several signalling cascades implicated in a myriad of cellular processes such as proliferation, differentiation, or apoptosis [6]. However, the exact mechanism of these ROS-mediated events is not fully understood. Studies have demonstrated that ROS activates receptor tyrosine kinases (RTK) such as the epidermal growth factor receptor (EGFR) and platelet-derived growth factor receptor (PDGFR), as well as their downstream kinases including PI3K/Akt and MAPK/ERK pathways leading to cell proliferation, nutrient uptake, and cell survival [7]. ROS can also activate several other kinases such as Protein Kinases C (PKCs), Protein Kinases G (PKGs), c-Jun N-terminal kinases (JNKs), p38 mitogen-activated protein kinases (p38 MAPKs) [8], and many more [9].

Disease-associated oxidative stress, due to the failure of the cell to maintain its redox homeostasis, may ultimately trigger unwanted cellular responses including programmed cell death or apoptosis [10]. Several genes and proteins including essential regulators of cell proliferation such as c-myc, ornithine decarboxylase (ODC), and PKC are affected during the above-mentioned processes [11,12,13,14,15,16,17]. ODC and c-myc play important roles in anabolic metabolism, DNA synthesis, regulation of gene expression, anti-differentiation, and cell growth [18,19,20,21,22,23]. ODC is involved in G1/S, S/G_2_, and G_2_/M cell cycle transitions [24]. On the other hand, c-myc plays a critical role in G_0/_S transition during the cell cycle progression. Downregulation of c-myc affects mitochondrial function, glycolysis and proliferation of endothelial cells, while its overexpression activates ODC which then promotes cell growth [25]. Importantly, ODC levels are tightly regulated as it is a potential oncogene whose overexpression contributes to cellular transformation by other oncogenes [26].

PKCs are serine/threonine kinases and belong to a protein family that encompasses 3 subfamilies: conventional PKCs, novel PKCs, and atypical PKCs. ROS covalently modify PKCs at their cysteine-rich zinc finger domain leading to their activation [27]. Interestingly, PKC activation can induce NADPH-oxidases (NOX) activity which further exacerbates ROS production [28]. Moreover, PKC activation is associated with cell survival and apoptosis, angiogenesis, extracellular matrix deposition, and contractility of vascular smooth muscle cells [17,29]. For instance, Wang et al. recently demonstrated that ROS-mediated apoptosis in cultured primary cardiomyocytes occurs through the activation of PKCβ [30]. This bidirectionality in PKC and NOX activation could have implications for cellular injury in various pathologies [31].

Endothelial cells (ECs) are crucial for the maintenance of cardiovascular homeostasis. Indeed, endothelial dysfunction is a critical step in the onset and progression of many cardiovascular diseases (CVDs) including atherosclerosis and hypertension [32,33]. Both the integrity and functions of the endothelial layer are greatly affected by ROS [34]. Furthermore, the interplay between ROS and PKC in endothelial function is well-established. For instance, overexpression of PKC increases intercellular adhesion molecule-1 expression, a pro-inflammatory protein, via ROS [35]. Moreover, PKC-mediated endothelial dysfunction involves both NOX and ROS production [36].

Dietary natural polyphenols such as those found in vegetables, tea, or coffee possess strong antioxidant capacities, and it is widely accepted that their consumption could preserve ECs function eventually reducing CVDs occurrence [37]. One of the most commonly consumed and studied polyphenols is resveratrol (RES). RES is a natural polyphenolic compound present in grapes, peanuts and some berries [38,39,40]. By virtue of its ability to reduce oxidative stress, cardiovascular protective effects of RES were proposed and later established in several in vitro, ex vivo, and animal studies [39,41]. However, the so far available clinical trials on humans have provided controversial results concerning the protective effects of RES against CVDs and its sequelae [42,43].

Several studies suggest that only lower concentrations of RES exert beneficial effects on ECs [44]. A biphasic concentration effect of RES has also been reported in angiogenesis assays [45]. Moreover, we and others have shown that high concentrations of RES increase rather than decrease ROS production [43,46,47,48,49]. More importantly, at high concentrations, RES acts as a xenobiotic compound whose metabolism produces elevated levels of ROS leading to cellular apoptosis. We have postulated that this ROS-mediated damage is through cytochrome P450, CYP2C9, metabolism of RES that produces excessive ROS which downregulates Akt—a key player in cell proliferation [43,46]. This observation is in contrast with the mainstream notion that increasing RES consumption will lead to more ROS scavenging and hence more cytoprotection.

Determining the optimal cytoprotective dose of RES has been hampered by the lack of a complete understanding of the molecular mechanisms underlying RES-induced pro-survival and pro-apoptotic effects. Therefore, with the aim of further dissecting the molecular basis of RES impact on EC homeostasis, here we assessed the expression of a series of genes (either at the mRNA or protein levels) involved in the regulation of cell survival and apoptosis such as *c-myc*, *ODC*, *Bcl-2*, *Bax,* and *Cyclin D1*. Moreover, based on the rational proposed in the introduction we also investigated (1) whether the potential bidirectionality between ROS and PKC is implicated in the biphasic response elicited by RES on ECs function, and (2) whether the assessed genes may be mechanistically involved in the above-hypothesized phenomenon.

## 2. Materials and Methods

Unless otherwise specified, all chemicals were purchased from Sigma (Sigma St. Louis, MO, USA)

### 2.1. Cell Culture and Treatment

Human umbilical vein endothelial cells (HUVECs) were obtained from Cell Applications (Cell Applications, San Diego, CA, USA) and cultured as previously described [43]. HUVECs were sub-cultured at a split ratio of 1:2 and used within three passages before cell viability was checked using trypan blue. Unless not otherwise specified, cells were plated in 96-well black plates (BD Falcon, Franklin Lakes, NJ) and processed for experiments in Medium M199 containing 5% FCS plus the different treatments. RES 10 mM stock solution was prepared in dimethyl sulfoxide (DMSO); final DMSO concentration in the diluted treatment media was less than 0.1%. DMSO (less than 0.1%) was used as a vehicle control. To investigate the involvement of ROS and NADPH oxidase in the RES-induced cellular effects, in selected experiments, cells were pretreated for 1 h with either 100 µM of the broad antioxidant Tempol [50] or with 10µM of the flavin-oxidase inhibitor Diphenyleneiodonium (DPI) [49,51,52]. The involvement of PKC in HUVECs response to RES treatment was investigated by using 2.5 µM of the broad PKC inhibitor chelerythrine (CHE) [53,54].

### 2.2. Measurement of Intracellular ROS

Intracellular ROS levels were assessed using the molecular probe 2′,7′-dichlorodihydrofluorescein diacetate ([H_2_DCF-DA]; Molecular Probe, Eugene, OR, USA), as previously described [55,56]. Within the cell, esterases cleave the acetate groups on H_2_DCF-DA, thus trapping the reduced form of the probe (H_2_DCF). Intracellular ROS oxidize H_2_DCF, yielding the fluorescent product, DCF. After treatments, cells were incubated for 30 min with Hanks′ Balanced Salt Solution (HBSS) containing 10 µM H_2_DCF-DA, then washed twice with HBSS and fluorescence was measured by using a GENios plus micro-plate reader (Tecan, Männedorf, CH). Excitation and emission wavelengths used for fluorescence quantification were 485 nm and 535 nm, respectively. All fluorescence measurements were corrected for background fluorescence and protein concentration. Using untreated cells as a reference, the anti- and pro-oxidant outcome was evaluated by comparison of five measurements and expressed as a percentage of untreated controls.

### 2.3. Determination of the Intracellular Redox State

**Figure 1:**
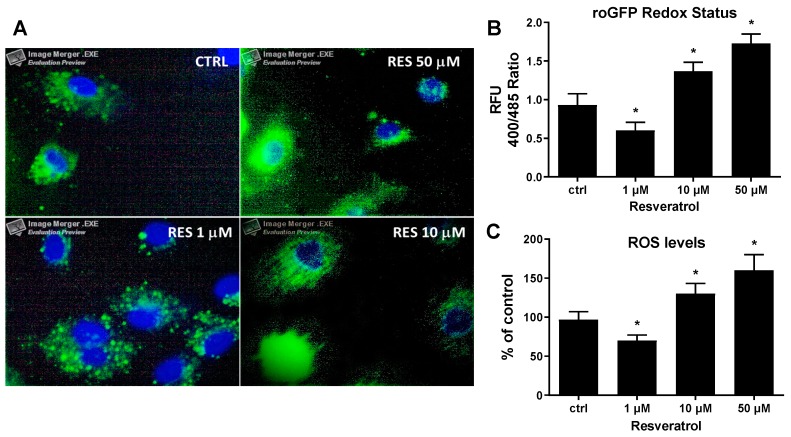
Concentration-dependent effect of resveratrol on HUVECs intracellular ROS levels and redox state. (**A**) Human umbilical vein endothelial cells (HUVECs) expressing the cytoplasmatic form of the redox-sensing green fluorescent protein (roGFP) were treated for 6 hrs in M199 medium with 5% FCS containing different concentrations of resveratrol (RES) as indicated. After treatment, cells were washed with PBS three times, fixed with 4% paraformaldehyde, counterstained with Hoechst, mounted and visualized by fluorescence microscopy. Images depict cytoplasmic merged photos of roGFP (green) and nuclear Hoechst staining (blue) of cells at 100X magnification. (**B**) HUVECs expressing the cytoplasmatic form of the roGFP were treated for 24 hrs with the indicated concentrations of resveratrol and then analyzed for the variation of the intracellular redox state as described in materials and methods. (**C**) HUVECs loaded with the ROS probe H_2_DCF-DA were treated for 6 hrs with the indicated concentrations of resveratrol then ROS levels were assessed as described in materials and methods. ctrl: untreated cells; and RFU: relative fluorescence units. Means ± SD of a representative experiment, performed in triplicates, are shown. *, significantly different from the control (*p <* 0.01).

The intracellular redox state was investigated by using the lentiviral vector encoding for the redox-sensing green fluorescent protein (roGFP), which reports the redox state of the GSH/GSSG pool in vivo in both plant and mammalian cells [57,58,59]. The procedure was performed as previously described [46,59,60]. Three days after virus infection, cultured cells showed a transduction efficiency of about 70% as depicted by Figure 1A, which is representative of six photos taken with a fluorescence microscope (Olympus XI70). roGFP has two fluorescence excitation maxima at 400 (oxidized form) and 485 nm (reduced form) and displays rapid and reversible ratiometric changes in fluorescence in response to changes in ambient redox potential and therefore the ratios of fluorescence from excitation at 400 and 485 nm indicate the extent of oxidation [58]. In place of confocal imaging analysis, we used a developed fluorometer-based method for monitoring roGFP oxidation [46,59,60,61]. Fluorescence measurements were performed in clear 24-well plates (Corning, Lowell, MA, USA) on a fluorescence plate reader GENios plus (Tecan, Männedorf, Zürich, Switzerland) from the upper side using multiple reads per well (the read pattern was square, and the number of reads was 2 x 2). Cells were excited using 400 and 485 nm filters, and fluorescence values were measured using a 535 nm emission filter. For background correction, emission intensities were determined for non-transformed cells (4 discs each experiment) exposed to the same excitation wavelengths under the same conditions. These values were averaged and subtracted from the fluorescence values of roGFP. The degree of oxidation of the roGFP was estimated from the ratios of light intensities obtained measuring 1-min intervals under 400 and 485 nm excitation. Treatment-induced variations of roGFP oxidation were estimated by comparison with roGFP oxidation in control untreated cells.

### 2.4. Determination of Cell Viability

Cell viability was assessed in 96-well plates (BD Falcon) by using the colorimetric 3-(4,5-dimethylthiazol-2-yl)-2,5- diphenyltetrazolium bromide (MTT reagent) assay (Promega, Madison, WI, USA) [51,62]. Yellow MTT reagent enters the cells and passes into the mitochondria where mitochondrial dehydrogenases of viable cells cleave the tetrazolium ring, yielding a reduced purple MTT formazan crystals which are insoluble in aqueous solutions. This reduction occurs only when mitochondrial enzymes are active, and therefore conversion can be directly related to the number of viable cells. The formazan crystals can be dissolved in acidified isopropanol. The resulting purple solution is spectrophotometrically measured at 570 nm. An increase in cell number results in a large amount of MTT formazan formed and an increase in absorbance at 570 nm. After 24 h of RES treatment, 20 µl of MTT solution (2 mg/mL) in medium M199 were added to the cells and incubated at 37 °C in a cell culture incubator for 2 h. At the end of the incubation period, the solution was removed, and the purple formazan product was solubilized with acidic isopropanol (0.04 N HCl in absolute isopropanol). Then plates were analyzed at 570 nm using a GENios plus micro-plate reader (Tecan). Results are expressed as a percent of untreated control cells.

### 2.5. Determination of DNA Synthesis

DNA synthesis was assessed by using a chemiluminescent immunoassay method, which is based on the measurement of BrdU incorporation during DNA synthesis (Cell Proliferation ELISA BrdU, Roche Applied Science). When cells are pulsed with BrdU, it is incorporated into newly synthesized DNA strands of actively proliferating cells. The incorporation of BrdU into cellular DNA can be detected using anti-BrdU antibodies, allowing assessment of the population of cells synthesizing DNA. Subconfluent HUVECs were treated for 24 hrs as indicated in the figure legends, and BrdU was added 12 hrs before the end of the experiments. After that, the culture supernatant was removed, and the cells were fixed with a Fix-Denat solution for 30 min. The Fix-Denat was discarded, and cells were incubated with an anti-BrdU antibody conjugated to horseradish peroxidase for 90 min. After rinsing three times with washing buffer, a peroxidase substrate solution was added and allowed to react for 3–10 min at room temperature. The horseradish peroxidase catalyzes the oxidation of diacyl hydrazide, where the reaction product decay from its excited state yields light. Finally, light emission was read by using a GENios Plus microplate reader (Tecan). Results were normalized for protein content and expressed as the mean ± SD of the relative fluorescence units (RFU) values [51,52,63]. Data are representative of three independent experiments and are shown as percent of untreated control cells.

### 2.6. Immunoblot Analysis

Immunoblotting analysis was performed as previously described [43,64,65]. Experiments were performed with subconfluent HUVECs in a T-25 culture flask (Falcon, Oxnard, CA, USA). After 12 hrs of RES treatment, the medium was removed, and cells were detached with 0.1% trypsin plus 0.02% EDTA in PBS, pH 7.3 and pelleted by centrifugation at 1,000x g for 5 min. The pellet was washed with PBS, centrifuged as above and then resuspended in 100 µl of a chilled lysis buffer (50 mM Hepes, pH 7.5, 150 mM NaCl, 1% Nonidet P-40, 0.5% sodium deoxycholate, 1 mM sodium vanadate, 50 mM sodium fluoride, 20 mM β-glycerophosphate, 0.1 mM okadaic acid, 1 mM phenylmethylsulfonyl fluoride, 20 mg/mL aprotinin, 50 mg/mL leupeptin, and 10 mM pepstatin). The samples were sonicated for 10 s (Branson, sonifer B-12, setting3) and incubated at 4 °C for 15 min, lysates were then centrifuged at 10,000× *g* for 15 min (4 °C) and analyzed for the protein content by Lowry method. Each sample was mixed with Laemmli sample buffer and boiled for 4 min. Equal amounts of proteins (10 to 20 µg/lane) were loaded. Sample and pre-stained molecular weight markers (Santa Cruz Biotechnology, Inc. Santa Cruz, CA, USA) were separated on 10% SDS-PAGE gel. Proteins were transferred to a nitrocellulose membrane in buffer containing 25 mM Tris-HCl, 192 mM glycine, and 20% methanol for 2 h at 350 mA at room temperature. Following transfer, nitrocellulose membranes were incubated for 1 h in 20 mM Tris-HCl, pH 7.6, 137 mM NaCl, 0.2% Tween 20 (TNT 20) with 5% non-fat dried milk, washed three-times in TNT 20 (3, 3, 5 min), and incubated for 1 h with primary antibody in TNT 20 containing 5% milk at room temperature. Cyclin D1, c-myc, and Bcl-2 proteins were detected using specific primary antibodies (Cell Signaling, Danvers, MA, USA). After further washing in TNT 20, membranes were incubated for 1 h with horseradish peroxidase-linked anti-IgG secondary antibody diluted 1:5000 (Bio-Rad, Hercules, CA, USA) and immunoreactive proteins were detected by ECL as described by the supplier (Amersham Pharmacia Biotech, Buckinghamshire, U.K). The intensities of bands were measured by using a Versadoc Imaging System (Bio-Rad). Band intensity was expressed as relative immunodensity units. The ratio between bands intensities of the loading control (β-actin) and the proteins of interest was calculated to normalize for potential variations in protein loading. Mean, and standard deviation, of four performed experiments was expressed as Relative immunodensity and was calculated after normalization to β-actin. Data are expressed as the mean ± SD.

### 2.7. Determination of DNA Fragmentation

Experiments were performed in T-25 culture flasks (Falcon, Oxnard, CA, USA) with subconfluent HUVECs. After RES treatment, the medium was removed, and cells were detached with 0.1% trypsin plus 0.02% EDTA in PBS, pH 7.3 and pelleted by centrifugation at 1,000x g for 5 min. The pellet was washed with PBS, centrifuged as above and then resuspended in 500 µl of detergent buffer (10 mM Tris [pH 7.4], 5 mM EDTA, 0.2% Triton). Pellet was vortexed for 2 min, incubated on ice for 30 min and then centrifugated at 27,000x g for 30 min. The supernatant was divided into two 250 µL aliquots, and 50 µL of ice-cold 5 M NaCl was added to each aliquot and the vortexed again. DNA was precipitated by adding 600 µL ethanol plus 150 µL 3 M sodium acetate at pH 5.2; then the solution was mixed by pipetting up and down. Tubes were incubated at −80 °C for 1 h. DNA was pelleted by 20 min of centrifugation at 20,000x g. After having carefully discarded the supernatant, DNA from different aliquots was pooled by re-dissolving the two pellets in a total of 400 µL extraction buffer (10 mM Tris and 5 mM EDTA). Two µL of 10 mg/mL DNase-free RNase was added, and samples were incubated for 5 h at 37 °C. Twenty-five µL proteinase K (20 mg/mL) and 40 µL of buffer (100 mM Tris pH 8.0, 100 mM EDTA, 250 mM NaCl) were added before incubating the sample overnight at 65 °C. DNA was extracted with phenol/chloroform/isoamyl alcohol (25:24:1) and precipitated with ethanol. After having carefully discarded the supernatant, the pellet was air-dried and resuspended in 20 µL Tris-acetate EDTA buffer supplemented with 2 µL of sample buffer (0.25% bromophenol blue, 30% glycerol). Then the DNA samples were separated electrophoretically on a 2% agarose gel containing 1 µg/mL ethidium bromide and visualized by ultraviolet transillumination. Band intensity was expressed as relative absorbance units. Intensities of the gel bands were measured by using a Versadoc Imaging System (Bio-Rad) and results were expressed as a percent of untreated control cells. [66,67,68]

### 2.8. Cell Cycle Analysis

Cell Cycle progression was monitored by quantification of cellular DNA content using the Tali^®^ Cell Cycle kit (Invitrogen) [69]. The analysis was performed according to the manufacturer′s instructions. Briefly, HUVECs were seeded at a density of 0.6 × 10^6^ cells/mL in 6-multiwell plate and treated with RES for 48 h. After harvesting by trypsinization, cells were rinsed with PBS then fixed in ice-cold 70% ethanol at −20 °C overnight. Following the centrifugation at 1,000x g for 5 min, cells were resuspended in PBS and centrifuged again at 500x g for 10 min. Cells were then stained in Tali^®^ Cell Cycle Solution (containing propidium iodide, RNase A, and Triton^®^ X-100) for 30-min, in the dark at RT. Data were analyzed using the Tali^®^ Image-Based Cytometer (Invitrogen) and the percentage of cells in each phase of cell cycle was determined using FACS Express Research Edition software (version 4.03; De Novo Software, NJ, USA) [70].

### 2.9. Semi-Quantitative Reverse Transcription-Polymerase Chain Reaction (RT-PCR)

RT-PCR: Experiments were performed in a T-25 culture flask (Falcon, Oxnard, CA, U.S.A.) with confluence contact-dependent growth inhibited cells. After 12 hrs of RES treatment, total RNA was extracted, reverse transcribed and amplified according to the procedure previously described [71,72,73]. One µg of total RNA from human endothelial cells was reverse-transcribed for 45 min at 37 °C. The reaction was performed in a solution of 25 µl containing, 50 mM Tris-HCl pH 8.3, 75 mM KCl, 3 mM MgCl_2_, 10 mM dithiothreitol (DTT), 0.2 mM of each dNTP, 0.1 µg of oligo dT, 200 units of M-MLV reverse transcriptase (Thermo Fisher Scientific, Waltham, Massachusetts, USA). The reaction mixture was then heated at 95 °C for 5 min to inactivate the enzyme. PCR amplification was performed in 25 µl of a reaction mixture containing: 5 µl of the reverse transcribed cDNA, 20 mM Tris-HCl pH 8.3, 50 mM KCl, 1.5 mM MgCl_2_, 2.5 units of Taq polymerase (Thermo Fisher Scientific), 0.2 mM of each dNTP, and 50 pmoles of each forward and reverse primer that had previously dissolved in TE solution (Tris 10 mM pH 8.0, EDTA 1 mM pH 8.0). Primers for *ODC*, *Bax*, *Bcl-2*, *c-myc*, and *GAPDH* were designed using Primer 3 software and optimized in the laboratory. Primers sequences were: Forward, 5′-CCACCCATGGCAAATTCCATGGCA-3′, reverse 5′-TCTAGACGGCAGGTCAGGTCCACC-3′ for GADPH; Forward,5′-TACCCTCTCAACGACAGCAGCTCGCCCAACTCCT-3′, Reverse,5′-TCTTGACATTCTCCTCGGTGTCCGAGGACCT-3′ for c-myc; Forward,5′-GCAGGATCCACCATGAACAACTTTGGTAA-3′, Reverse,5′-GCCGAGATCTCAGAAGAAGAAACTTC-3′ for ODC; Forward, 5′-CCTTTTCTACTTTGCCAGCAAAC-3′, Reverse, 5′-GAGGCCGTCCCAACCAC-3′ for BAX; Forward, 5′-AGGGTCAGATGGACACATGGTG-3′, Reverse, 5′-CGTTGCCTGTGGGTGACTAATC-3′. *Bcl-2*. *ODC* and *GAPDH* were amplified in 30 cycles (ODC: 72 °C for 60 s, 95 °C for 40 s, 43 °C for 40 s; *GAPDH*: 95 °C for 30 s, 58 °C for 30 s, 72 °C for 1 min); while Bax and c-myc were amplified in 35 cycles (*Bax*: 95 °C for 45 s, 60 °C for 50 s, 72 °C for 60 s; c-myc: 72 °C for 60 s, 95 °C for 40 s, 60 °C for 40 s); *Bcl-2* was amplified in 38 cycles (95 °C for 45 s, 68 °C for 50 s, 72 °C for 60 s). The number of amplification cycles was determined experimentally for each primer pair and to find out the exponential phase of amplification, different PCR cycles from 15 to 50 were tested. Using 30 PCR cycles, all the PCR products were within the linear phase of the reaction. Similar conditions have been previously reported for semiquantitative analysis of gene expression [71,72,73].

Gel Electrophoresis: The PCR products were loaded onto Ethidium Bromide stained 1% agarose gels in TBE. A 1 kbp DNA ladder molecular weight marker (Gibco BRL, Paisley, U.K) was run on every gel to confirm the expected molecular weight of the amplification product. GAPDH was used for each sample as an internal control for mRNA integrity and equal loading.

Acquisition of gel images and quantitative analysis: Images of the RT-PCR ethidium bromide-stained agarose gels were acquired and densitometrically analyzed by using the Versadoc Imaging System (Bio-Rad). Band intensity was expressed as relative absorbance units. The ratio between the specific gene and GAPDH was calculated to normalize to initial variations in sample concentration and as a control for reaction efficiency. Mean and standard deviation of all experiments performed was calculated after normalization to GAPDH [71,72,73].

### 2.10. Determination of PKC Activity

HUVECs cultured in T-25 culture flasks (Falcon) were treated with RES for 40 min, then cells were processed as previously described [65]. After RES treatment, the medium was removed, and cells were detached with 0.1% trypsin plus 0.02% EDTA in PBS, pH 7.3 and pelleted by centrifugation, at 1000× *g* for 5 min. The pellet was washed with PBS, centrifuged and then resuspended in 100 µl of a chilled lysis buffer (50 mM HEPES, pH 7.5, 150 mM NaCl, 1% Nonidet P-40, 0.5% sodium deoxycholate, 1 mM sodium vanadate, 50 mM sodium fluoride, 20 mM β-glycerophosphate, 0.1 mM okadaic acid, 1 mM phenylmethylsulfonyl fluoride, 20 mg/mL aprotinin, 50 mg/mL leupeptin, and 10 mM pepstatin). Samples were sonicated for 10 s (Branson, sonifer B-12, setting3) and incubated at 4 °C for 15 min; lysates were then centrifuged at 10,000× *g* for 15 min (4 °C) and analyzed for the protein content by Lowry method. Aliquots of the homogenate were taken to measure total PKC activity by a PKC enzyme assay system (PepTag Non-Radioactive Protein Kinase Assay; Promega, Madison, WI, USA), according manufacturer instructions. The PepTag^®^ Non-Radioactive Protein Kinase Assays provide a rapid, sensitive and non-radioactive method to detect cAMP-dependent protein kinase (PKA) and protein kinase C (PKC) activity. The PepTag^®^ Assays use brightly colored, fluorescent peptide substrates that are highly specific for the kinases in question. Phosphorylation by PKC or PKA alters the peptide substrate net charge, allowing phosphorylated and non-phosphorylated versions of the substrate to be rapidly separated on an agarose gel. Using the PepTag^®^ Assay, less than 10 ng of kinase can be detected in under 2 h. Two micrograms of PepTagTMC1 peptide (PKC-specific peptide) were incubated with the 20 µg of the protein samples in a final volume of 25 µL for 30 min at 30 °C. The reactions were stopped by heating to 95 °C for 10 min. The samples were loaded on a 0.8% agarose gel and run at 100 Volts for 15 min. Phosphorylated peptide migrated towards the anode, while non-phosphorylated peptide migrated towards the cathode. Intensities of charged phosphorylated bands were measured by using a Versadoc Imaging System (Bio-Rad). Band intensity was expressed as relative absorbance units. The positive control was run to verify the Kinase activity and results were expressed as a percent of untreated control cells [74].

### 2.11. Statistical Analysis

Data are expressed as means ± S.D. of three different experiments. One-way analysis of variance (ANOVA) followed by a post-hoc Newman-Keuls Multiple Comparison Test was used to detect differences of means among treatments with significance defined as *p* < 0.05. When appropriate, two-way ANOVA with a Bonferoni post-test was used to assess any differences among the treatments and the times (*p* < 0.05). Statistical analysis was performed using GraphPad Prism version 5.00 for Windows (GraphPad Software, San Diego California USA).

## 3. Results

To demonstrate the biphasic effect of RES concentrations on ROS production, HUVECs loaded with the ROS probe H_2_DCF-DA were treated with different concentrations of RES (1–50 μM). As compared to untreated cells, low RES concentration (1μM) decreases basal intracellular ROS levels, while higher concentrations (10µM and 50μM) augment ROS levels (Figure 1C). To confirm this observation, the variation of intracellular ROS level in response to RES was further investigated by assessing the cellular redox state. To this end, HUVECs were transduced with the redox state sensor roGFP, which fluoresces when oxidized, and treated with RES as indicated in the legend of Figure 1. RES at the concentration of 1 μM exerted a significant reduction of the roGFP oxidation state as compared with ro-GFP-infected untreated control cells, thus RES at 1 μM effectively acts as an antioxidant agent (Figure 1A,B). However, when roGFP-infected HUVECs were exposed to higher RES levels (50 μM), an increase of the roGFP oxidation state was elicited (Figure 1A,B).

**Figure 2:**
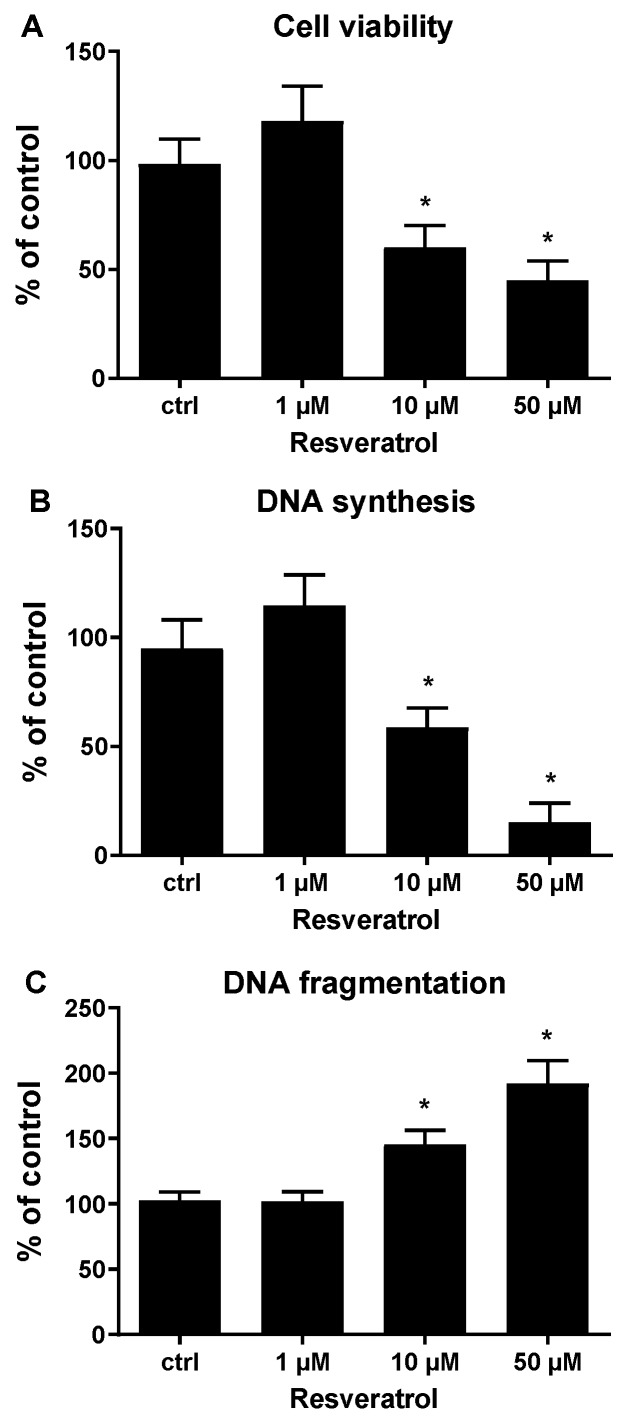
Resveratrol concentration-dependently affect HUVECs survival, DNA synthesis, and apoptosis. (**A**) Sub-confluent HUVECs were grown for 24 h in M199 medium with 5% FCS, containing the indicated concentrations of resveratrol. Subsequently, cells were assessed for the viability as reported in materials and methods. (**B**) Sub-confluent HUVECs were grown for 24 h in M199 medium with 5% FCS, containing the indicated concentrations of resveratrol. Subsequently, cells were assessed for the DNA synthesis as reported in material and methods. (**C**) Sub-confluent HUVECs were grown for 24 h in M199 medium with 5% FCS, containing the indicated concentrations of Resveratrol. Subsequently, cells were assessed for the DNA fragmentation as reported in materias and methods. ctrl: untreated cells. Data represent means ± SDs of a representative experiment, performed in triplicates. *, significantly different from the control (*p <* 0.01).

Accordingly, the effect of RES on HUVECs viability was tested. To this end, HUVECs were treated with increasing concentrations (1–50 μM) of RES, and the viability of the cells was detected using the MTT assay. Figure 2A illustrates that lower concentrations of RES (1 μM) elicited a non-significant increase in cell viability, while higher RES concentrations (10 and 50 μM) caused a significant decrease in cell viability, with less cell viability seen at 50 μM. To evaluate if RES has an impact on cell viability would transpire in the context of cell proliferation, the influence of different RES concentrations on HUVECs DNA synthesis was studied. Cells were treated with increasing concentrations of RES (1–50 μM), then DNA synthesis was assessed using BrdU incorporation assay. At low concentrations (1 μM), RES provoked a non-significant increase in DNA synthesis (Figure 2B). However, higher RES concentrations (10 and 50 μM), significantly inhibited DNA synthesis, with higher inhibition observed at 50 μM (Figure 2B). Consistent with the observed pro-oxidant effect, the increased RES dosage elicited a corresponding decrease in both cell survival and DNA synthesis, suggesting a strong correlation between the pro-oxidant effect and the cell damage exerted by RES. Furthermore, the observed inhibited DNA synthesis rates under higher concentrations of RES can partially explain the decreased cell viability with 10 and 50 μM RES.

Increased generation of ROS usually culminates in apoptotic cell death. An increased apoptosis rate can also contribute to the decreased cell viability seen with high RES concentrations. In order to determine if lower cell viability under high RES concentrations is solely due to inhibited cell proliferation, apoptosis levels were compared under varying RES concentrations. The degree of DNA fragmentation in HUVECs exposed to increasing concentrations of RES, reflective of apoptosis levels, was determined. As shown in Figure 2C, lower RES concentrations exhibit no differences in DNA fragmentation as compared to control untreated HUVECs, on the other hand, higher concentrations of RES (10 and 50 μM), show significantly increased DNA fragmentation, especially at 50 μM RES. The results obtained in Figure 2, indicate that the lower cell viability observed using higher RES concentrations is due to both an anti-proliferative effect and a pro-apoptotic effect exerted by high concentrations of RES on HUVECs. These results may also indicate that the anti-proliferative effect of high concentrations of RES may be correlated with the facilitation of an apoptotic program.

**Figure 3:**
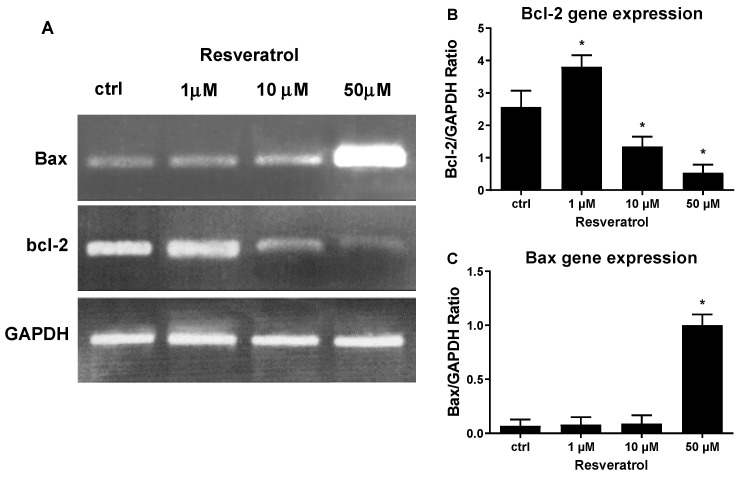
High concentrations of RES modulate gene expression of apoptotic genes. HUVECs grown in M199 medium with 5% FCS were stimulated for 40 min with different concentrations of RES as indicated. (**A**) Representative agarose electrophoretic gel reporting the PCR product size in base pairs for Bax 291; *Bcl-2* 460 and GAPDH 598. PCR product semiquantitative analysis for the *Bcl-2* (**B**) and Bax (**C**) in HUVECs treated as described. ctrl: untreated cells. Values are Means ± SD of a representative experiment, performed in triplicates. *, significantly different from the control (*p <* 0.01).

To gain molecular insight into the mechanism of high concentration RES induced apoptosis, we quantified the mRNA levels of the anti-apoptotic gene, *Bcl-2*, and the pro-apoptotic gene, *Bax*. To this end, HUVECs were treated with 1 μM, 10 μM, and 50 μM of RES and RT-PCR was performed to determine the expression levels of both genes. As shown in Figure 3B, the concentration of 1 μM RES significantly increased *Bcl-2* mRNA levels, which could explain the increased viability of HUVECs obtained at the same concentration (Figure 2). As compared to control cells, higher RES concentrations significantly diminished *Bcl-2* mRNA levels, reaching the highest decrease at 50 μM (Figure 3B). A different effect was exerted by RES on Bax, which showed a low expression at lower RES concentration (1 μM), while its mRNA levels spiked at higher RES concentrations (50 μM) (Figure 3C). Figure 3, which illustrates and compares Bax and *Bcl-2* gene expression under different RES concentrations, confirms the role RES plays in the induction of cellular apoptosis observed with higher RES concentrations and further attests to the biphasic effect exerted by different RES concentrations.

**Figure 4:**
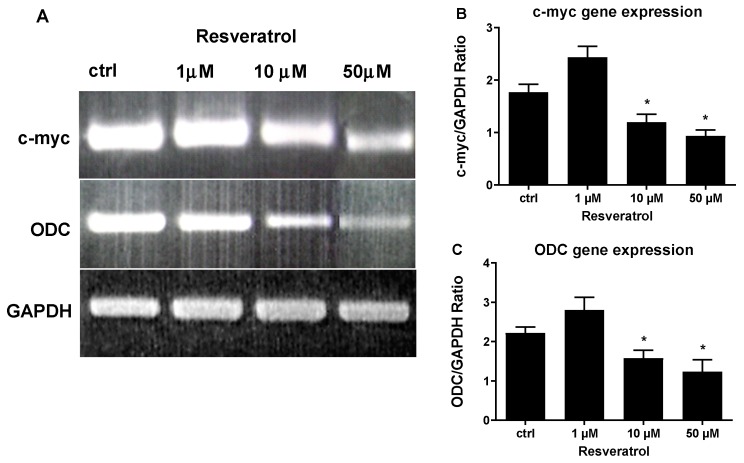
High concentrations of RES modulate gene expression of prosurvival genes. HUVECs grown in M199 medium with 5% FCS were stimulated for 40 min with different concentrations of RES as indicated. (**A**) Representative agarose electrophoretic gel reporting the PCR product size in base pairs for c-myc 479; ODC 120 and GAPDH 598. PCR product semiquantitative analysis for the *c-myc* (**B**) and *ODC* (**C**) in HUVECs treated as described. ctrl: untreated cells. Values are Means ± SD of a representative experiment, performed in triplicates. *, significantly different from the control (*p <* 0.01).

For a more molecular characterization of the RES induced inhibition of DNA synthesis observed in Figure 2, gene expression of c-myc and ODC was studied by RT-PCR. c-myc and ODC are genes that play pivotal roles in cell cycle progression and cell proliferation and are tightly controlled throughout the cell cycle. RT-PCR of c-myc and ODC was performed on total RNA from HUVECs treated with increasing concentrations of RES. c-myc gene expression was significantly increased by 1 μM RES treatment, and ODC gene expression was also increased by the same treatment although non-significantly (Figure 4A,B). These data further corroborate and explain the results obtained in Figure 3, showing a low concentration of RES activating the antiapoptotic gene *Bcl-2*. Figure 4 also indicates that higher concentrations of RES (10 and 50 μM) significantly decreased the mRNA levels of the prosurvival genes c-myc and ODC. Again, these data go hand in hand with the cell survival data obtained in Figure 2. RES, also, has a dichotomous effect on gene expression of c-myc and ODC.

**Figure 5:**
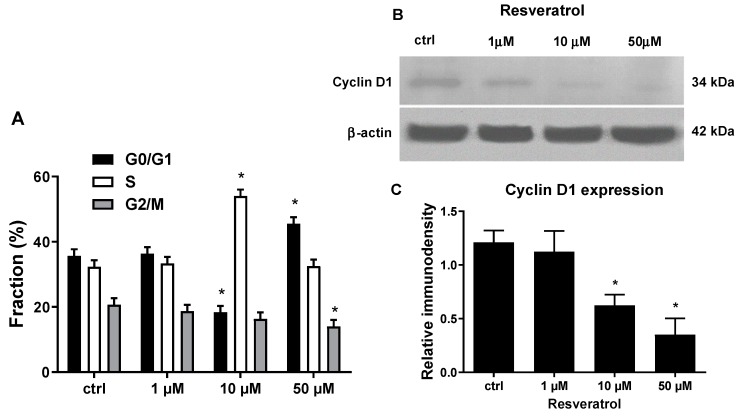
RES biphasically affects HUVECs cell cycle progression and cyclin D1 protein expression. (**A**) Sub-confluent HUVECs were grown for 48 h in M199 medium with 5% FCS, containing the indicated concentrations of resveratrol. Subsequently, cells were assessed for their cell cycle distribution as reported in materials and methods. (**B**,**C**) Sub-confluent HUVECs grown in M199 medium containing 5% FCS were stimulated for 12 hrs with different concentrations of RES as indicated. (**B**) Representative immunoblot of cyclin D and β-actin of HUVECs treated with different RES concentrations. (**C**) The graph represents the immunodensity quantitative analysis of three different immunoblot experiments. ctrl: untreated cells. Mean ± SD values of a representative experiment, performed in triplicates, are shown. *, significantly different from the control (*p <* 0.01).

Among the investigated genes affected by RES, c-myc and Bcl-2 are reported to be essential regulators of cell cycle progression, an effect at least in part mediated by their interaction with the cell cycle regulator protein cyclin D1 from G_0_/G1 to S phase [75,76,77,78]. That given, we further dissected the effect of RES on cell proliferation by investigating the effect of different RES concentrations on HUVECs cell cycle progression machinery. Data in Figure 5A clearly indicate that a high concentration of RES blocked HUVECs cell cycle progression by prompting their accumulation in the G_0_/G1 or S phase depending on the dosage. Cyclin D1 protein expression was assessed, since it controls G_0_/G1 to S phase transition, by western blotting. As shown in Figure 5B,C, at a lower concentration of RES (1 µM) there is no difference in cyclin D1 protein levels as compared to control cells, but at higher concentrations (10 and 50 µM), RES significantly inhibits cyclin D1 protein expression. This finding strongly supports the hypothesis that RES, by modulating c-myc and Bcl-2 expression, may suppress HUVECs proliferation by downregulating cyclin D1 expression ultimately arresting the cells in G0/S phases of the cell cycle. Moreover, the observed decrease in cyclin D1 protein levels also corroborates to the low cell viability observed in Figure 2 and further confirm the biphasic effect of RES on essential genes involved cell cycle progression and apoptosis.

**Figure 6:**
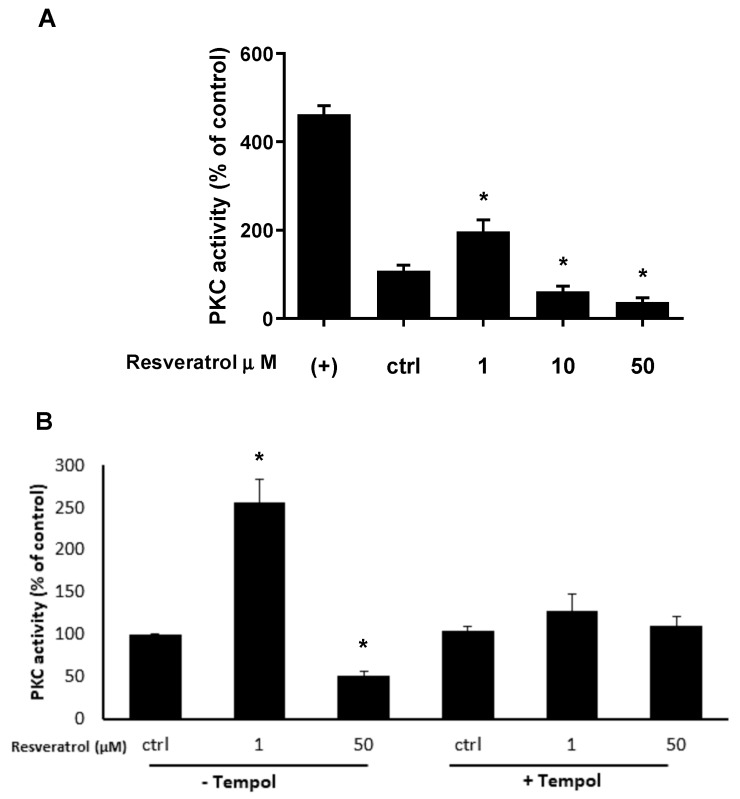
(**A**) RES generated ROS biphasically modulates PKC activity. HUVECs were grown in M199 medium containing 5% FCS and then treated for 40 min with the indicated concentrations of RES and then PKC activity was measured as described in the materials and methods section. (**B**) Same as in A, but in the absence or presence of Tempol. HUVECs were pre-treated or not for 1 hr with the ROS scavenger Tempol (100 µM). PKC activities are expressed as % of control (ctrl). (+): positive control of PKC activation (phosphatidylserine 0.2 mg/mL). Means ± SDs of a representative experiment, performed in triplicates, are shown. *, significantly different from the control (*p <* 0.01).

We have previously reported that RES affects HUVECs survival by eliciting Flavin-oxidase mediated ROS generation which in turn inhibits Akt activation ultimately prompting cell death by apoptosis [43]. However, we have no clue concerning the fact that ROS generation, in RES-induced endothelial cells, may act on enzymes upstream of Akt such as PKC for one instance [79,80]. In this regard, a bidirectional link has been proposed between PKC activation and ROS generation in different cell type including endothelial cells [17,28,30,35,36]. We hypothesized that RES-induced ROS generation might biphasically modulates PKC activity, leading to the activation of pro- or anti-survival machinery depending on the RES dosage. To address this hypothesis, we assessed the activity of PKC in HUVECs treated with different RES concentrations in the absence or presence of the ROS scavenger Tempol. Figure 6A illustrates that RES at low concentrations (1 μM) enhances PKC activity, while at higher RES concentrations (25 and 50 μM), PKC activity is abrogated, clearly indicating that different RES concentrations exert a biphasic effect on PKC activation.

To test the involvement of ROS in the RES biphasic activation of PKC, different RES concentrations were used to modulate PKC activity in the presence and absence of the ROS scavenger Tempol [81,82,83]. Comparing the effect of low concentration RES (1 µM) in the presence and absence of Tempol, it can be seen that, the scavenger action of Tempol reverses the increased PKC activation elicited by low RES concentration (Figure 6B). The same figure also shows that at high RES concentrations (50 µM), PKC activity is diminished, but this RES effect is abolished when cells are pre-treated with Tempol. These data strongly indicate that the biphasic effect of RES on PKCs activation is dependent on the level of generated ROS.

**Figure 7:**
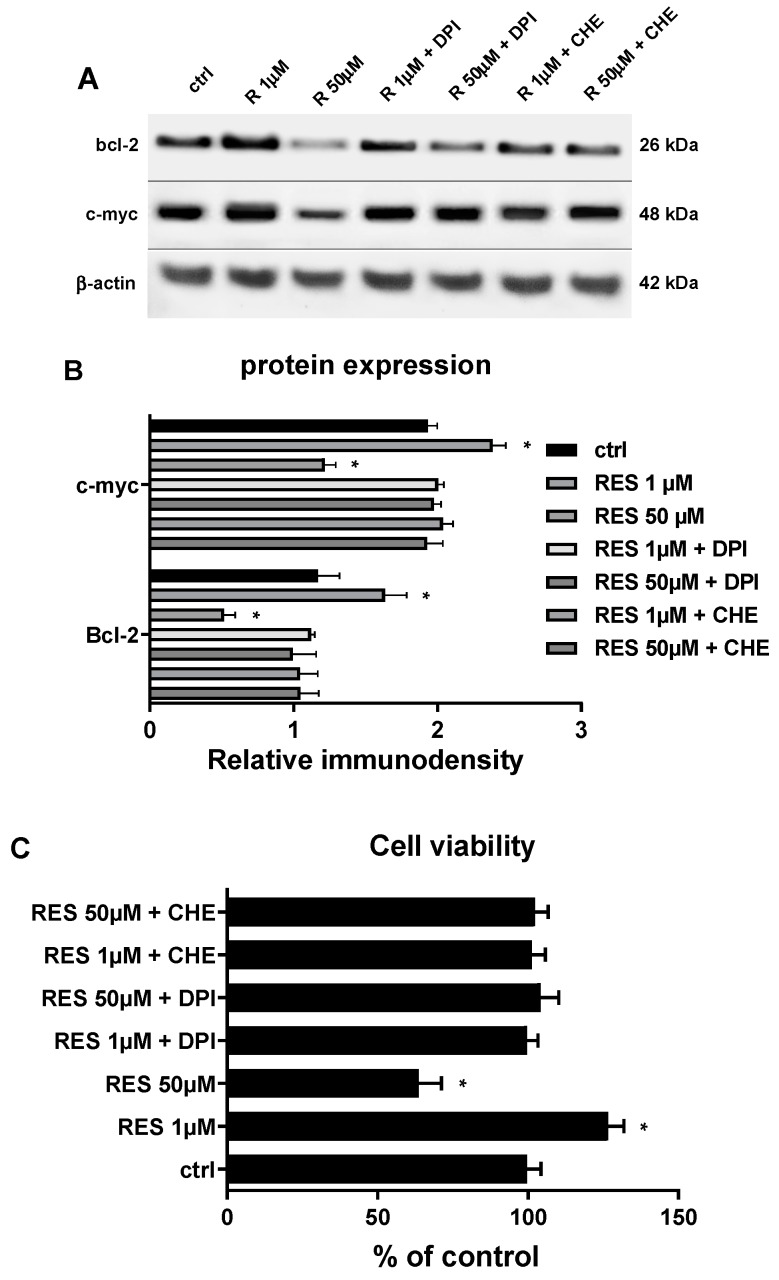
(**A**,**B**) Flavin oxidase-generating enzymes and PKC mediated RES effect c-myc and Bcl-2 protein expression in HUVECs. Sub-confluent HUVECs grown in M199 medium containing 5% FCS were stimulated for 12 hrs with different concentrations of RES as indicated. HUVECs were pre-treated for 1 hr with the flavin oxidase inhibitor Diphenyleneiodonium (DPI) or with the PKC inhibitor chelerythrine (CHE). The figure depicts a representative immunoblot of Bcl-2, c-myc and β-actin. (**B**) A graph representing the quantitative analysis of the immunodensity of three different immunoblot experiments. (**C**) As in A and B, but measuring HUVECs viability. Cells were assessed for the viability as reported in materias and methods. CTRL: untreated cells. Means ± SD of a representative experiment, performed in triplicates, are shown. *, significantly different from the control (*p <* 0.01).

If the effect of RES on PKC is mediated by ROS it is reasonable to hypothesize that both ROS and PKC could be involved in modulating the expression of c-myc and Bcl-2, ultimately defining the cell fate based on the RES dosage. Therefore, by using a broad flavin oxidase inhibitor, DPI, and a broad PKC inhibitor, CHE, we tested whether flavin oxidase-mediated ROS generation and PKC activation are involved in modulating c-myc and Bcl-2 protein expression and thus HUVECs proliferation in response to RES treatment. As reported in Figure 7, both DPI and CHE were able to abrogate the biphasic effect of RES on c-myc and Bcl-2 gene expression and HUVECs proliferation. These data strongly implicate these two enzymes in the cellular response to different RES dosages.

## 4. Discussion

Resveratrol, 3,5,4′ trihydroxystilbene, is a phytoalexin that is naturally produced by plants in response to infection. This naturally occurring compound possesses antioxidant beneficial cardioprotective, anti-cancerous and anti-inflammatory properties [84]. However, the effects of resveratrol have been mainly tested against oxidative stress-induced damage or other toxic insults, whereas relatively few studies have been performed to understand RES potential effects under normal conditions. For example, RES at a concentration of 50 µM, a dosage that under our current experimental conditions induces HUVEC damage, protects cardiomyocytes against anoxia/reoxygenation-elicited impairment [85] and protects HUVECs from ox-LDL-induced oxidative damage [86]. In this regard, it has been suggested that RES can work as a pro-oxidant under low redox conditions, while it acts as an antioxidant under highly oxidative conditions [49,87]. Such behavior has been reported for other natural antioxidants in different experimental models [88], suggesting that the interaction of exogenous antioxidants depends on the cellular redox state, and is essential especially when precise redox modulation is needed to determinate whether the final cell fate is survival or death. Previous reports have shown that RES, at low concentrations, acts as an antioxidant; however, at higher concentrations, it becomes a pro-oxidant that increases ROS generation and initiates apoptosis and cell senescence [89]. In endothelial cells with low oxidative stress, high RES concentrations are harmful rather than beneficial [49]. RES may act as an anti-oxidant and scavenges the low levels of ROS, which are required for ROS physiological signalling. Under these conditions, the antioxidant effects of RES are not needed, and the cell eliminates RES using the anti-xenobiotic metabolic pathway which generates high ROS levels [46]. We have previously demonstrated that ROS generation leads to mitochondria-dependent apoptotic cell death [46]. In fact, RES, at high concentrations, caused cell damage through CYP-mediated increase of ROS levels. CYP2C9, involved in xenobiotic metabolism and a key source of intracellular ROS, metabolizes RES producing excessive ROS levels. In addition, drugs, toxins or pathological conditions that increase ROS production promote the loss of mitochondrial membrane potential (MMP), leading to cell death. Treatment of cells with high concentrations of RES and CsA, a mitochondrial permeability transition pore inhibitor, prevented MMP and cell death by ROS, linking high levels of RES to mitochondrial damage and mitochondrial induced cell death [46].

This biphasic RES concentration effect is further supported by Pasciu et al. who showed that instead of counteracting ROS effect on endothelial cells, RES actually induced ROS production [43]. Furthermore, prolonged RES exposure leads to senescence and cell cycle arrest due to increased and prolonged ROS production [90]. Indeed, the biphasic RES concentration effect on ROS production has been demonstrated by us and others [43,45,46,47,48,63]. In this work, we further dissected at a molecular level this RES functional dichotomy on ROS production, where high levels of RES induced ROS production and produced an oxidized cell-redox state and low RES concentrations inhibited ROS generation. This biphasic RES effect on ROS production transpired into a biphasic RES effect on apoptotic rate, DNA synthesis, and PKC activity in HUVECs. In fact, high RES levels enhanced apoptosis, inhibited DNA synthesis, and inhibited PKC activity in HUVECS, while low RES dosage inhibited apoptosis, enhanced DNA synthesis, and increased PKC activity in HUVECs. These data show that RES functional dichotomy affects cell physiology differently, where cell survival is enhanced at low RES levels and suppressed at high RES levels.

The molecular mechanisms by which RES can biphasically act on endothelial cells and induce apoptosis are still controversial and undefined. This work was undertaken to investigate these molecular mechanisms and gain more understanding concerning the impact of RES on HUVECs. In this respect, we found that lower RES concentrations of 1 μM, increase DNA synthesis and expression of genes involved in cell cycle regulation (c-myc, ODC, and cyclin D1) and inhibits apoptosis and decreases the expression of pro-apoptotic genes (Bax) and increases the expression of anti-apoptotic genes (Bcl-2). However, at higher concentrations (10 and 50 μM), RES enhances apoptosis and pro-apoptotic Bax gene expression and decreases DNA synthesis and the expression of c-myc, ODC, cyclin-D1, and the anti-apoptotic Bcl-2 gene. The dichotomous effect of RES on Bcl-2 and Bax expression is consistent with the biphasic RES modulation of ROS, and thereafter modulation of apoptosis. Bcl-2 and Bax regulate apoptosis at the level of the mitochondria [91], here we find these new data to further support our previous report that high RES concentrations do induce high ROS levels that then elicit mitochondrial induced cell death [46]. In fact, Bax acts by creating pores in the mitochondrial outer membrane which then leads to the release of mitochondrial intermembrane space proteins such as cytochrome-c and disruption of MMP [91]. This role of Bax is also consistent with our previous work in which we show that high RES concentrations modulate MMP [46]. Nevertheless, it should be mentioned that Bax activation can also contribute to ROS generation [91], so there remains the testable possibility that RES activates Bax which then initiates apoptosis and ROS generation. As we only have data concerning Bcl-2, future work should focus if RES biphasically also affects Bax at the translational levels and if there is a biphasic modulation of downstream mediators of mitochondrial-induced apoptosis such as the release of cytochrome-c and the consequent activation of PARP and effector caspases. Above all, future research should address if Bcl-2 and Bax are required at all for MMP alteration and the execution of the cell death program initiated by high RES concentrations.

c-myc regulates the transition from G_0_ to S phase in the cell cycle, and ODC can act at G_1_/S, S/G_2_ and G2/M cell cycle transition [24]. Noteworthy, c-myc over expression activates ODC which then promotes cell growth [25]. At the same time, c-myc is known to have pro and anti-apoptotic roles [22]. In addition, there is a bidirectional interplay between c-myc and ROS. c-myc overexpression can upregulate ROS production [23], and ROS can inhibit c-myc expression [12]. Interestingly, c-myc downregulation is a pre-requisite for ROS induced apoptosis in M14 human melanoma cells [92], which is evident in the current study where high RES concentrations have downregulated both c-myc mRNA levels and protein expression and increased apoptosis. More importantly, both Bcl-2 and c-myc are an essential regulator of cell cycle progression, an effect at least in part mediated by their interaction with the cell cycle regulator protein cyclin D1. In fact, while Bcl-2 overexpression induces cyclin D1 promoter activity, its silencing exerts a decrease in cyclin D1 expression [75,76]. On the other hand, c-myc has been proposed as one of the main drivers in promoting Cyclin D1 expression by linking mitogen-stimulated signal transduction to the cell cycle [77,78]. Consonant with what is discussed above are our data in Figure 5, showing the downregulation of cyclin D1 paralleled by the cell cycle arrest in G0/S phase. Summation of all the previous discussion makes us envisage molecular pathways for the biphasic action of RES.

A possible pathway is that at high RES concentrations, xenobiotic metabolism of RES by CYP2C9 leads to elevated ROS generation, which then induces a change in c-myc and Bcl-2 levels that then, by affecting Cyclin D1 expression, modulate cell cycle progression and apoptosis. Of course, c-myc down-regulation can be a cause or a result of high ROS levels, but this remains to be determined. High ROS levels, in the presence of low c-myc levels, can also alter MMP (probably through Bax) leading to mitochondrial activation of apoptosis. In this scenario, Bax also can be acting upstream or down stream of ROS, and this again remains to be determined. Although an inhibitory effect of RES on PKC has been reported in adenocarcinoma cells [93], we show for the first time that RES has a biphasic concentration effect on PKC activation in normal human endothelial cells and demonstrated the implication of ROS in its mediation. Higher concentrations of RES downregulate PKC activity, while low RES concentrations enhanced PKC activity (Figure 6A). In this regard, we believe that ROS can both activate or inhibit PKC depending on ROS localization, source, and concentration [94,95]. Indeed, high RES concentrations can induce high levels of ROS that inhibit PKC, while low RES concentrations generate lower levels of ROS that elicit, instead, the activation of PKC (Figure 6A). This biphasic effect of RES appears to be dependent on ROS levels since the addition of a ROS scavenger, Tempol, ablated it (Figure 6B). In fact, on one hand, Tempol is able to abrogate the PKC inhibition elicited by the high levels of ROS produced by 50 µM RES. On the other hand, Tempol suppresses PKC activity induced by low levels of ROS, which in turn are produced by low RES concentrations. (Figure 6B). Here we can see an interplay between ROS and PKC. Consistently, many studies have reported that there is a bidirectional crosstalk between ROS and PKC activation [28,30,96,97,98,99,100,101,102]. Figure 6B also points to the fact that ROS production precedes PKC action and it maybe the point connecting ROS-generating flavin oxidases enzymes such as CYP2C9 or NOX with the cell cycle machinery. In fact, failure of RES to induce its biphasic effect in the presence of the flavin inhibitor DPI implicated flavin oxidase-generated ROS as the mechanistic link between RES and the cell cycle machinery (Figure 7A). The same figure also indicates the involvement of PKC in the RES effect upon cell cycle regulatory genes and HUVECs viability as pointed out by the ability the PKC inhibitor CHE to significantly abrogate them. The findings reported in Figure 6B, along with those of Figure 7 suggest that PKC may be located downstream CYP2C9 and/or NOX and being modulated biphasically based on the ROS produced by different RES dosages.

In this regard, it will be interesting to perform the reverse experiment where pan-PKC activity is inhibited, and ROS production is monitored. This experiment will further demonstrate the bidirectional interplay between ROS and PKC, and RES for that effect. All the above can be modeled into a signaling pathway where high RES concentrations increase ROS production that activate PKC which then triggers a pathway that down-regulates the genes and proteins involved in cell proliferation (c-myc, ODC, and cyclin D1) and that abrogates genes and proteins involved in cell apoptosis (Bax and Bcl-2), thus inducing a cell cycle arrest and initiating mitochondrial apoptosis as discussed above. The reverse can take place at low RES levels, leading to enhanced cell survival and lower apoptotic cell death. In this pathway, ROS signaling to PKC could be dependent on NOXs activation since NADPH oxidases rather than CYPs are more likely involved in the signaling between ROS and PKC [28,98]. In this regard, micromolar concentrations of RES (0,1–0,5 µM) have been reported to activate Akt, increase NO production and inhibit NADPH oxidase-dependent ROS generation in human platelets [103], which seems a common property possessed by others NA such as quercetin and red wine polyphenols [104,105]. Although the above data are strongly consistent with our present results, testing this notion under our experimental conditions, require specific NOXs inhibitors or knockdown experiments to better dissect the cellular source of ROS modulated by different RES concentrations. This functional dichotomy is not a property of RES per se but is intimately tied to a general ROS signaling mechanism. For instance, physiologic production of ROS is known to activate the JAK/STAT pathway, while dramatically increased ROS cause its inhibition [9]. Similarly, physiological levels of ROS promote cell survival under hypoxic/ischemic conditions, but higher levels can precipitate cell death via apoptotic or autophagic pathways [9].

It remains to be determined, whether the concentration-dependent effect of RES always involves ROS generation as a general biological process, or other molecular pathways are implicated [89,106]. For instance, RES inhibits adipocyte differentiation and induces apoptosis in pre-adipocytes and mature adipocytes at concentrations beyond 20 μM [107,108,109], while RES works as an antidiabetic drug promoting glucose uptake in the range of 0.01–1 μM [110]. RES-also elicited pro- and anti-fibroti responses of human tubular epithelial cell in vitro, and in mice kidneys in vivo are dependent on the RES concentration employed [111]. Although many RES-elicited responses, including some of those mentioned above, are mediated by sirtuins [108,109,110,112,113], a link between ROS, Sirtuin and RES has been reported only during its protective action in oxidative-induced experimental models [114,115,116]. Future studies should address if such a link exists between RES, sirtuins and ROS generation in HUVECs and other types of cells under both normal and oxidative stress conditions.

The study of the negative impact of high RES concentrations on endothelial cell function should be further investigated to more deeply dissect the molecular underpinnings behind high dosage effects, both in vitro and in vivo. Moreover, the public should be made aware that antioxidants supplementation is still an area of investigation, and that potential health benefits can be attained only at specific natural antioxidants dosages that are yet to be identified.

## References

[1] Foyer C.H., Noctor G. (2005). Redox homeostasis and antioxidant signaling: A metabolic interface between stress perception and physiological responses. Plant Cell.

[2] Turrens J.F. (2013). Reactive oxygen species. Encyclopedia of Biophysics.

[3] Schieber M., Chandel N.S. (2014). Ros function in redox signaling and oxidative stress. Curr. Biol..

[4] Clarke T.B. (2014). Microbial programming of systemic innate immunity and resistance to infection. PLoS PATHOG..

[5] Kamata H., Hirata H. (1999). Redox regulation of cellular signalling. Cell Signal.

[6] Ray P.D., Huang B.W., Tsuji Y. (2012). Reactive oxygen species (ros) homeostasis and redox regulation in cellular signaling. Cell Signal.

[7] Cantley L.C. (2002). The phosphoinositide 3-kinase pathway. Science.

[8] Shinkai Y., Iwamoto N., Miura T., Ishii T., Cho A.K., Kumagai Y. (2012). Redox cycling of 1,2-naphthoquinone by thioredoxin1 through cys32 and cys35 causes inhibition of its catalytic activity and activation of ask1/p38 signaling. Chem. Res. Toxicol..

[9] Krylatov A.V., Maslov L.N., Voronkov N.S., Boshchenko A.A., Popov S.V., Gomez L., Wang H., Jaggi A.S., Downey J.M. (2018). Reactive oxygen species as intracellular signaling molecules in the cardiovascular system. Curr. Cardiol. Rev..

[10] Sinha K., Das J., Pal P.B., Sil P.C. (2013). Oxidative stress: The mitochondria-dependent and mitochondria-independent pathways of apoptosis. Archiv. Toxicol..

[11] Lan L., Wei W., Zheng Y., Niu L., Chen X., Huang D., Gao Y., Mo S., Lu J., Guo M. (2018). Deferoxamine suppresses esophageal squamous cell carcinoma cell growth via erk1/2 mediated mitochondrial dysfunction. Cancer Lett..

[12] Wu G., Liu T., Li H., Li Y., Li D., Li W. (2018). C-myc and reactive oxygen species play roles in tetrandrine-induced leukemia differentiation. Cell Death Dis..

[13] Sander C.S., Chang H., Hamm F., Elsner P., Thiele J.J. (2004). Role of oxidative stress and the antioxidant network in cutaneous carcinogenesis. Int. J. Dermatol..

[14] Huang C., Hsu P., Hung Y., Liao Y., Liu C., Hour C., Kao M., Tsay G.J., Hung H., Liu G.Y. (2005). Ornithine decarboxylase prevents methotrexate-induced apoptosis by reducing intracellular reactive oxygen species production. Apoptosis Int. J. Program. Cell Death.

[15] Klann E., Roberson E.D., Knapp L.T., Sweatt J.D. (1998). A role for superoxide in protein kinase c activation and induction of long-term potentiation. J. Biol. Chem..

[16] Knapp L.T., Klann E. (2002). Potentiation of hippocampal synaptic transmission by superoxide requires the oxidative activation of protein kinase c. J. Neurosci.Off. J. Soc. Neurosc..

[17] Cosentino-Gomes D., Rocco-Machado N., Meyer-Fernandes J.R. (2012). Cell signaling through protein kinase c oxidation and activation. Int. J. Mol. Sci..

[18] Moshier J.A., Malecka-Panas E., Geng H., Dosescu J., Tureaud J., Skunca M., Majumdar A.P. (1995). Ornithine decarboxylase transformation of nih/3t3 cells is mediated by altered epidermal growth factor receptor activity. Cancer Res..

[19] Thomas T., Thomas T.J. (2001). Polyamines in cell growth and cell death: Molecular mechanisms and therapeutic applications. Cell. Mol. Life Sci. CMLS.

[20] Desbarats L., Schneider A., Muller D., Burgin A., Eilers M. (1996). Myc: A single gene controls both proliferation and apoptosis in mammalian cells. Experientia.

[21] Dang C.V. (2013). Myc, metabolism, cell growth, and tumorigenesis. Cold Spring Harb. Perspect. Med..

[22] Meyer N., Penn L.Z. (2008). Reflecting on 25 years with myc. Nat. Rev. Cancer.

[23] Vafa O., Wade M., Kern S., Beeche M., Pandita T.K., Hampton G.M., Wahl G.M. (2002). C-myc can induce DNA damage, increase reactive oxygen species, and mitigate p53 function: A mechanism for oncogene-induced genetic instability. Mol. Cell.

[24] He W., Roh E., Yao K., Liu K., Meng X., Liu F., Wang P., Bode A.M., Dong Z. (2017). Targeting ornithine decarboxylase (odc) inhibits esophageal squamous cell carcinoma progression. NPJ Precis. Oncol..

[25] Baudino T.A., McKay C., Pendeville-Samain H., Nilsson J.A., Maclean K.H., White E.L., Davis A.C., Ihle J.N., Cleveland J.L. (2002). C-myc is essential for vasculogenesis and angiogenesis during development and tumor progression. Genes Dev..

[26] Hibshoosh H., Johnson M., Weinstein I.B. (1991). Effects of overexpression of ornithine decarboxylase (odc) on growth control and oncogene-induced cell transformation. Oncogene.

[27] Steinberg S.F. (2015). Mechanisms for redox-regulation of protein kinase c. Front. Pharmacol..

[28] Thallas-Bonke V., Jha J.C., Gray S.P., Barit D., Haller H., Schmidt H.H., Coughlan M.T., Cooper M.E., Forbes J.M., Jandeleit-Dahm K.A. (2014). Nox-4 deletion reduces oxidative stress and injury by pkc-alpha-associated mechanisms in diabetic nephropathy. Physiol. Rep..

[29] Gao X., Schottker B. (2017). Reduction-oxidation pathways involved in cancer development: A systematic review of literature reviews. Oncotarget.

[30] Wang Y., Zhao J., Yang W., Bi Y., Chi J., Tian J., Li W. (2015). High-dose alcohol induces reactive oxygen species-mediated apoptosis via pkc-beta/p66shc in mouse primary cardiomyocytes. Biochem. Biophys. Res. Commun..

[31] Jha J.C., Banal C., Okabe J., Gray S.P., Hettige T., Chow B.S.M., Thallas-Bonke V., De Vos L., Holterman C.E., Coughlan M.T. (2017). Nadph oxidase nox5 accelerates renal injury in diabetic nephropathy. Diabetes.

[32] Gammone M.A., Riccioni G., D′Orazio N. (2015). Marine carotenoids against oxidative stress: Effects on human health. Mar. Drugs.

[33] Cai H., Harrison D.G. (2000). Endothelial dysfunction in cardiovascular diseases: The role of oxidant stress. Circ. Res..

[34] Knock G.A., Ward J.P. (2011). Redox regulation of protein kinases as a modulator of vascular function. Antioxid. Redox Signal..

[35] Joo H.K., Lee Y.R., Choi S., Park M.S., Kang G., Kim C.S., Jeon B.H. (2017). Protein kinase c beta ii upregulates intercellular adhesion molecule-1 via mitochondrial activation in cultured endothelial cells. Korean J. Physiol. Pharmacol..

[36] Gao L., Mann G.E. (2009). Vascular nad(p)h oxidase activation in diabetes: A double-edged sword in redox signalling. Cardiovasc. Res..

[37] Sisein E.A. (2014). Biochemistry of free radicals and antioxidants. Sch. Acad. J. Biosci..

[38] Baur J.A., Sinclair D.A. (2006). Therapeutic potential of resveratrol: The in vivo evidence. Nat. Rev.Drug Dis..

[39] Li H., Xia N., Förstermann U. (2012). Cardiovascular effects and molecular targets of resveratrol. Nitric Oxide.

[40] Pervaiz S., Holme A.L. (2009). Resveratrol: Its biologic targets and functional activity. Antioxid. Redox Signal..

[41] Ladurner A., Schachner D., Schueller K., Pignitter M., Heiss E.H., Somoza V., Dirsch V.M. (2014). Impact of trans-resveratrol-sulfates and -glucuronides on endothelial nitric oxide synthase activity, nitric oxide release and intracellular reactive oxygen species. Molecules.

[42] Willcox B.J., Curb J.D., Rodriguez B.L. (2008). Antioxidants in cardiovascular health and disease: Key lessons from epidemiologic studies. Am. J. Cardiol..

[43] Pasciu V., Posadino A.M., Cossu A., Sanna B., Tadolini B., Gaspa L., Marchisio A., Dessole S., Capobianco G., Pintus G. (2010). Akt downregulation by flavin oxidase–induced ros generation mediates dose-dependent endothelial cell damage elicited by natural antioxidants. Toxicol. Sci..

[44] Wong R.H., Nealon R.S., Scholey A., Howe P.R. (2016). Low dose resveratrol improves cerebrovascular function in type 2 diabetes mellitus. Nutr. Metabol. Cardiovasc. Dis. NMCD.

[45] Wang H., Zhou H., Zou Y., Liu Q., Guo C., Gao G., Shao C., Gong Y. (2010). Resveratrol modulates angiogenesis through the gsk3beta/beta-catenin/tcf-dependent pathway in human endothelial cells. Biochem. Pharmacol..

[46] Posadino A.M., Cossu A., Giordo R., Zinellu A., Sotgia S., Vardeu A., Hoa P.T., Nguyen le H.V., Carru C., Pintus G. (2015). Resveratrol alters human endothelial cells redox state and causes mitochondrial-dependent cell death. Food Chem. Toxicol..

[47] Heo J.R., Kim S.M., Hwang K.A., Kang J.H., Choi K.C. (2018). Resveratrol induced reactive oxygen species and endoplasmic reticulum stressmediated apoptosis, and cell cycle arrest in the a375sm malignant melanoma cell line. Int. J. Mol. Med..

[48] Ji S., Zheng Z., Liu S., Ren G., Gao J., Zhang Y., Li G. (2018). Resveratrol promotes oxidative stress to drive dlc1 mediated cellular senescence in cancer cells. Exp. Cell Res..

[49] Giordo R., Cossu A., Pasciu V., Hoa P.T., Posadino A.M., Pintus G. (2013). Different redox response elicited by naturally occurring antioxidants in human endothelial cells. Open Biochem. J..

[50] Perez-Cremades D., Bueno-Beti C., Garcia-Gimenez J.L., Ibanez-Cabellos J.S., Hermenegildo C., Pallardo F.V., Novella S. (2017). Extracellular histones disarrange vasoactive mediators release through a cox-nos interaction in human endothelial cells. J. Cell. Mol. Med..

[51] Posadino A.M., Phu H.T., Cossu A., Giordo R., Fois M., Thuan D.T.B., Piga A., Sotgia S., Zinellu A., Carru C. (2017). Oxidative stress-induced akt downregulation mediates green tea toxicity towards prostate cancer cells. Toxicol. In Vitro.

[52] Fois A.G., Posadino A.M., Giordo R., Cossu A., Agouni A., Rizk N.M., Pirina P., Carru C., Zinellu A., Pintus G. (2018). Antioxidant activity mediates pirfenidone antifibrotic effects in human pulmonary vascular smooth muscle cells exposed to sera of idiopathic pulmonary fibrosis patients. Oxid. Med. Cell. Longev..

[53] Pintus G., Tadolini B., Maioli M., Posadino A.M., Gaspa L., Ventura C. (1999). Heparin down-regulates the phorbol ester-induced protein kinase c gene expression in human endothelial cells: Enzyme-mediated autoregulation of protein kinase c-alpha and -delta genes. FEBS Lett..

[54] Pintus G., Tadolini B., Maioli M., Posadino A.M., Bennardini F., Bettuzzi S., Ventura C. (1998). Heparin inhibits phorbol ester-induced ornithine decarboxylase gene expression in endothelial cells. FEBS Lett..

[55] Boin F., Erre G.L., Posadino A.M., Cossu A., Giordo R., Spinetti G., Passiu G., Emanueli C., Pintus G. (2014). Oxidative stress-dependent activation of collagen synthesis is induced in human pulmonary smooth muscle cells by sera from patients with scleroderma-associated pulmonary hypertension. Orphanet J. Rare Dis..

[56] Posadino A.M., Porcu M.C., Marongiu B., Cossu A., Piras A., Porcedda S., Falconieri D., Cappuccinelli R., Biosa G., Pintus G. (2012). Antioxidant activity of supercritical carbon dioxide extracts of salvia desoleana on two human endothelial cell models. Food Res. Int..

[57] Meyer A.J., Brach T., Marty L., Kreye S., Rouhier N., Jacquot J.P., Hell R. (2007). Redox-sensitive gfp in arabidopsis thaliana is a quantitative biosensor for the redox potential of the cellular glutathione redox buffer. Plant J..

[58] Dooley C.T., Dore T.M., Hanson G.T., Jackson W.C., Remington S.J., Tsien R.Y. (2004). Imaging dynamic redox changes in mammalian cells with green fluorescent protein indicators. J. Biol. Chem..

[59] Cossu A., Posadino A.M., Giordo R., Emanueli C., Sanguinetti A.M., Piscopo A., Poiana M., Capobianco G., Piga A., Pintus G. (2012). Apricot melanoidins prevent oxidative endothelial cell death by counteracting mitochondrial oxidation and membrane depolarization. PLoS ONE.

[60] Posadino A.M., Cossu A., Piga A., Madrau M.A., Del Caro A., Colombino M., Paglietti B., Rubino S., Iaccarino C., Crosio C. (2011). Prune melanoidins protect against oxidative stress and endothelial cell death. Front. Biosci. (Elite Ed).

[61] Rosenwasser S., Rot I., Meyer A.J., Feldman L., Jiang K., Friedman H. (2010). A fluorometer-based method for monitoring oxidation of redox-sensitive gfp (rogfp) during development and extended dark stress. Physiol. Plant..

[62] Posadino A.M., Biosa G., Zayed H., Abou-Saleh H., Cossu A., Nasrallah G.K., Giordo R., Pagnozzi D., Porcu M.C., Pretti L. (2018). Protective effect of cyclically pressurized solid(-)liquid extraction polyphenols from cagnulari grape pomace on oxidative endothelial cell death. Molecules.

[63] Posadino A.M., Cossu A., Giordo R., Zinellu A., Sotgia S., Vardeu A., Hoa P.T., Deiana L., Carru C., Pintus G. (2013). Coumaric acid induces mitochondrial damage and oxidative-mediated cell death of human endothelial cells. Cardiovasc. Toxicol..

[64] Debidda M., Sanna B., Cossu A., Posadino A.M., Tadolini B., Ventura C., Pintus G. (2003). Nami-a inhibits the pma-induced odc gene expression in ecv304 cells: Involvement of pkc/raf/mek/erk signalling pathway. Int. J. Oncol..

[65] Pintus G., Tadolini B., Posadino A.M., Sanna B., Debidda M., Carru C., Deiana L., Ventura C. (2003). Pkc/raf/mek/erk signaling pathway modulates native-ldl-induced e2f-1 gene expression and endothelial cell proliferation. Cardiovasc. Res..

[66] Sanna B., Debidda M., Pintus G., Tadolini B., Posadino A.M., Bennardini F., Sava G., Ventura C. (2002). The anti-metastatic agent imidazolium trans-imidazoledimethylsulfoxide-tetrachlororuthenate induces endothelial cell apoptosis by inhibiting the mitogen-activated protein kinase/extracellular signal-regulated kinase signaling pathway. Arch. Biochem. Biophys..

[67] Kasibhatla S., Amarante-Mendes G.P., Finucane D., Brunner T., Bossy-Wetzel E., Green D.R. (2006). Analysis of DNA fragmentation using agarose gel electrophoresis. Cold Spring Harb. Protoc..

[68] Rahbar Saadat Y., Saeidi N., Zununi Vahed S., Barzegari A., Barar J. (2015). An update to DNA ladder assay for apoptosis detection. Bioimpacts.

[69] Pozarowski P., Darzynkiewicz Z. (2004). Analysis of cell cycle by flow cytometry. Checkpoint Controls and Cancer.

[70] Klimaszewska-Wisniewska A., Halas-Wisniewska M., Tadrowski T., Gagat M., Grzanka D., Grzanka A. (2016). Paclitaxel and the dietary flavonoid fisetin: A synergistic combination that induces mitotic catastrophe and autophagic cell death in a549 non-small cell lung cancer cells. Cancer Cell Int..

[71] Marone M., Mozzetti S., De Ritis D., Pierelli L., Scambia G. (2001). Semiquantitative rt-pcr analysis to assess the expression levels of multiple transcripts from the same sample. Biol. Proced. Online.

[72] Cheng H., Pan Y., Yao Y., Zhu Z., Chen J., Sun X., Qiu Y., Ding Y. (2017). Expression and significance of caveolin-1 in hepatitis b virus-associated hepatocellular carcinoma. Exp. Ther. Med..

[73] Li H., Spagnol G., Zheng L., Stauch K.L., Sorgen P.L. (2016). Regulation of connexin43 function and expression by tyrosine kinase 2. J. Biol. Chem..

[74] Shao B., Bayraktutan U. (2013). Hyperglycaemia promotes cerebral barrier dysfunction through activation of protein kinase c-β. Diab. Obes. Metabol..

[75] Lin H.M., Lee Y.J., Li G., Pestell R.G., Kim H.R. (2001). Bcl-2 induces cyclin d1 promoter activity in human breast epithelial cells independent of cell anchorage. Cell Death Differ..

[76] Tucker C.A., Kapanen A.I., Chikh G., Hoffman B.G., Kyle A.H., Wilson I.M., Masin D., Gascoyne R.D., Bally M., Klasa R.J. (2008). Silencing bcl-2 in models of mantle cell lymphoma is associated with decreases in cyclin d1, nuclear factor-κb, p53, bax, and p27 levels. Mol. Cancer Ther..

[77] Yu Q., Ciemerych M.A., Sicinski P. (2005). Ras and myc can drive oncogenic cell proliferation through individual d-cyclins. Oncogene.

[78] Daksis J.I., Lu R.Y., Facchini L.M., Marhin W.W., Penn L.J. (1994). Myc induces cyclin d1 expression in the absence of de novo protein synthesis and links mitogen-stimulated signal transduction to the cell cycle. Oncogene.

[79] Gliki G., Wheeler-Jones C., Zachary I. (2002). Vascular endothelial growth factor induces protein kinase c (pkc)-dependent akt/pkb activation and phosphatidylinositol 3′-kinase-mediates pkc delta phosphorylation: Role of pkc in angiogenesis. Cell Biol. Int..

[80] Aronis K.N., Chamberland J.P., Mantzoros C.S. (2013). Glp-1 promotes angiogenesis in human endothelial cells in a dose-dependent manner, through the akt, src and pkc pathways. Metabolism.

[81] Marciniak A., Walczyna B., Rajtar G., Marciniak S., Wojtak A., Lasiecka K. (2016). Tempol, a membrane-permeable radical scavenger, exhibits anti-inflammatory and cardioprotective effects in the cerulein-induced pancreatitis rat model. Oxid. Med. Cell. Longev..

[82] Bernardy C.C.F., Zarpelon A.C., Pinho-Ribeiro F.A., Calixto-Campos C., Carvalho T.T., Fattori V., Borghi S.M., Casagrande R., Verri W.A. (2017). Tempol, a superoxide dismutase mimetic agent, inhibits superoxide anion-induced inflammatory pain in mice. Biomed. Res. Int..

[83] Batinic-Haberle I., Reboucas J.S., Spasojevic I. (2010). Superoxide dismutase mimics: Chemistry, pharmacology, and therapeutic potential. Antioxid. Redox Signal..

[84] Carrizzo A., Forte M., Damato A., Trimarco V., Salzano F., Bartolo M., Maciag A., Puca A.A., Vecchione C. (2013). Antioxidant effects of resveratrol in cardiovascular, cerebral and metabolic diseases. Food Chem. Toxicol. Int. J. Pub. Br. Indust. Biol. Res. Associat..

[85] Tian M., Xie Y., Meng Y., Ma W., Tong Z., Yang X., Lai S., Zhou Y., He M., Liao Z. (2019). Resveratrol protects cardiomyocytes against anoxia/reoxygenation via dephosphorylation of vdac1 by akt-gsk3 beta pathway. Eur. J. Pharmacol..

[86] Lefevre J., Michaud S.-E., Haddad P., Dussault S., Ménard C., Groleau J., Turgeon J., Rivard A. (2007). Moderate consumption of red wine (cabernet sauvignon) improves ischemia-induced neovascularization in apoe-deficient mice: Effect on endothelial progenitor cells and nitric oxide. FASEB J..

[87] Gadacha W., Ben-Attia M., Bonnefont-Rousselot D., Aouani E., Ghanem-Boughanmi N., Touitou Y. (2009). Resveratrol opposite effects on rat tissue lipoperoxidation: Pro-oxidant during day-time and antioxidant at night. Redox Rep..

[88] Kontush A., Finckh B., Karten B., Kohlschütter A., Beisiegel U. (1996). Antioxidant and prooxidant activity of alpha-tocopherol in human plasma and low density lipoprotein. J. Lipid Res..

[89] Mukherjee S., Dudley J.I., Das D.K. (2010). Dose-dependency of resveratrol in providing health benefits. Dose Response.

[90] Schilder Y., Heiss E., Schachner D., Ziegler J., Reznicek G., Sorescu D., Dirsch V. (2009). Nadph oxidases 1 and 4 mediate cellular senescence induced by resveratrol in human endothelial cells. Free Radic. Biol. Med..

[91] Gross A., McDonnell J.M., Korsmeyer S.J. (1999). Bcl-2 family members and the mitochondria in apoptosis. Genes Dev..

[92] Biroccio A., Benassi B., Amodei S., Gabellini C., Del Bufalo D., Zupi G. (2001). C-myc down-regulation increases susceptibility to cisplatin through reactive oxygen species-mediated apoptosis in m14 human melanoma cells. Mol. Pharmacol..

[93] Atten M.J., Attar B.M., Milson T., Holian O. (2001). Resveratrol-induced inactivation of human gastric adenocarcinoma cells through a protein kinase c-mediated mechanism. Biochem. Pharmacol..

[94] Jiang F., Zhang Y., Dusting G.J. (2011). Nadph oxidase-mediated redox signaling: Roles in cellular stress response, stress tolerance, and tissue repair. Pharmacol. Rev..

[95] Giorgi C., Agnoletto C., Baldini C., Bononi A., Bonora M., Marchi S., Missiroli S., Patergnani S., Poletti F., Rimessi A. (2010). Redox control of protein kinase c: Cell- and disease-specific aspects. Antioxid. Redox Signal..

[96] Bouwman R.A., Musters R.J., van Beek-Harmsen B.J., de Lange J.J., Boer C. (2004). Reactive oxygen species precede protein kinase c-delta activation independent of adenosine triphosphate-sensitive mitochondrial channel opening in sevoflurane-induced cardioprotection. Anesthesiology.

[97] Inoguchi T., Sonta T., Tsubouchi H., Etoh T., Kakimoto M., Sonoda N., Sato N., Sekiguchi N., Kobayashi K., Sumimoto H. (2003). Protein kinase c-dependent increase in reactive oxygen species (ros) production in vascular tissues of diabetes: Role of vascular nad(p)h oxidase. J. Am. Soc. Nephrol..

[98] Stein J., Steven S., Bros M., Sudowe S., Hausding M., Oelze M., Munzel T., Grabbe S., Reske-Kunz A., Daiber A. (2017). Role of protein kinase c and nox2-derived reactive oxygen species formation in the activation and maturation of dendritic cells by phorbol ester and lipopolysaccharide. Oxidat. Med. Cell. Longev..

[99] Gray R.D., Lucas C.D., MacKellar A., Li F., Hiersemenzel K., Haslett C., Davidson D.J., Rossi A.G. (2013). Activation of conventional protein kinase c (pkc) is critical in the generation of human neutrophil extracellular traps. J. Inflamm. (London, England).

[100] Liou J.S., Chen C.Y., Chen J.S., Faller D.V. (2000). Oncogenic ras mediates apoptosis in response to protein kinase c inhibition through the generation of reactive oxygen species. J. Biol. Chem..

[101] Wu W.S., Tsai R.K., Chang C.H., Wang S., Wu J.R., Chang Y.X. (2006). Reactive oxygen species mediated sustained activation of protein kinase c alpha and extracellular signal-regulated kinase for migration of human hepatoma cell hepg2. Mol. Cancer Res. MCR.

[102] Lee H.B., Yu M.R., Song J.S., Ha H. (2004). Reactive oxygen species amplify protein kinase c signaling in high glucose-induced fibronectin expression by human peritoneal mesothelial cells. Kidney Int..

[103] Gresele P., Pignatelli P., Guglielmini G., Carnevale R., Mezzasoma A.M., Ghiselli A., Momi S., Violi F. (2008). Resveratrol, at concentrations attainable with moderate wine consumption, stimulates human platelet nitric oxide production. J. Nutr..

[104] Sanchez M., Galisteo M., Vera R., Villar I.C., Zarzuelo A., Tamargo J., Perez-Vizcaino F., Duarte J. (2006). Quercetin downregulates nadph oxidase, increases enos activity and prevents endothelial dysfunction in spontaneously hypertensive rats. J. Hypertens..

[105] Lopez-Sepulveda R., Jimenez R., Romero M., Zarzuelo M.J., Sanchez M., Gomez-Guzman M., Vargas F., O′Valle F., Zarzuelo A., Perez-Vizcaino F. (2008). Wine polyphenols improve endothelial function in large vessels of female spontaneously hypertensive rats. Hypertension.

[106] Salehi B., Mishra A.P., Nigam M., Sener B., Kilic M., Sharifi-Rad M., Fokou P.V.T., Martins N., Sharifi-Rad J. (2018). Resveratrol: A double-edged sword in health benefits. Biomedicines.

[107] Chen S., Zhou N., Zhang Z., Li W., Zhu W. (2015). Resveratrol induces cell apoptosis in adipocytes via ampk activation. Biochem. Biophys. Res. Commun..

[108] Chen S., Xiao X., Feng X., Li W., Zhou N., Zheng L., Sun Y., Zhang Z., Zhu W. (2012). Resveratrol induces sirt1-dependent apoptosis in 3t3-l1 preadipocytes by activating ampk and suppressing akt activity and survivin expression. J. Nutr. Biochem..

[109] Chen S., Li Z., Li W., Shan Z., Zhu W. (2011). Resveratrol inhibits cell differentiation in 3t3-l1 adipocytes via activation of ampk. Can. J. Physiol. Pharmacol..

[110] Chen S., Zhao Z., Ke L., Li Z., Li W., Zhang Z., Zhou Y., Feng X., Zhu W. (2018). Resveratrol improves glucose uptake in insulin-resistant adipocytes via sirt1. J. Nutr. Biochem..

[111] Liu S., Zhao M., Zhou Y., Wang C., Yuan Y., Li L., Bresette W., Chen Y., Cheng J., Lu Y. (2019). Resveratrol exerts dose-dependent anti-fibrotic or pro-fibrotic effects in kidneys: A potential risk to individuals with impaired kidney function. Phytomedicine.

[112] Wong R.H.X., Howe P.R.C. (2018). Resveratrol counteracts insulin resistance-potential role of the circulation. Nutrients.

[113] Wicinski M., Socha M., Walczak M., Wodkiewicz E., Malinowski B., Rewerski S., Gorski K., Pawlak-Osinska K. (2018). Beneficial effects of resveratrol administration-focus on potential biochemical mechanisms in cardiovascular conditions. Nutrients.

[114] Bagul P.K., Deepthi N., Sultana R., Banerjee S.K. (2015). Resveratrol ameliorates cardiac oxidative stress in diabetes through deacetylation of nfkb-p65 and histone 3. J. Nutr. Biochem..

[115] Zhao L., Wang Y., Wang Z., Xu Z., Zhang Q., Yin M. (2015). Effects of dietary resveratrol on excess-iron-induced bone loss via antioxidative character. J. Nutr. Biochem..

[116] Curro M., Trovato-Salinaro A., Gugliandolo A., Koverech G., Lodato F., Caccamo D., Calabrese V., Ientile R. (2015). Resveratrol protects against homocysteine-induced cell damage via cell stress response in neuroblastoma cells. J. Neurosci. Res..

